# The Effects of Early Prenatal Alcohol Exposure on Epigenome and Embryonic Development

**DOI:** 10.3390/genes12071095

**Published:** 2021-07-19

**Authors:** Essi Wallén, Pauliina Auvinen, Nina Kaminen-Ahola

**Affiliations:** Environmental Epigenetics Laboratory, Department of Medical and Clinical Genetics, Medicum, University of Helsinki, 00290 Helsinki, Finland; essi.wallen@helsinki.fi (E.W.); pauliina.auvinen@helsinki.fi (P.A.)

**Keywords:** embryonic stem cells, environmental epigenetics, epigenetic reprogramming, DNA methylation, fetal alcohol spectrum disorders, histone modifications, miRNAs, mouse models

## Abstract

Prenatal alcohol exposure is one of the most significant causes of developmental disability in the Western world. Maternal alcohol consumption during pregnancy leads to an increased risk of neurological deficits and developmental abnormalities in the fetus. Over the past decade, several human and animal studies have demonstrated that alcohol causes alterations in epigenetic marks, including DNA methylation, histone modifications, and non-coding RNAs. There is an increasing amount of evidence that early pregnancy is a sensitive period for environmental-induced epigenetic changes. It is a dynamic period of epigenetic reprogramming, cell divisions, and DNA replication and, therefore, a particularly interesting period to study the molecular changes caused by alcohol exposure as well as the etiology of alcohol-induced developmental disorders. This article will review the current knowledge about the in vivo and in vitro effects of alcohol exposure on the epigenome, gene regulation, and the phenotype during the first weeks of pregnancy.

## 1. Introduction

### 1.1. Consequences of PAE

Prenatal alcohol exposure (PAE) is the underlying cause for a variety of birth defects referred to as Fetal Alcohol Spectrum Disorders (FASD). It is a non-diagnostic umbrella term for all the alcohol-related developmental disorders and birth defects, including four diagnostics categories [1]. The most severe form is known as fetal alcohol syndrome (FAS) with diagnosed growth retardation, central nervous system neurodevelopmental deficits, and craniofacial dysmorphology [2,3,4]. After the identification of FAS, it became clear that not all prenatally alcohol exposed individuals expressed all the features of FAS. Alcohol-associated disabilities represent a spectrum, consisting of growth deficits from mild to severe, physical abnormalities, neurocognitive, and behavioral deficits, as well as an increased vulnerability to mental health problems and other comorbidities [5,6,7]. In addition to FAS, there are the following three other diagnostic categories: partial fetal alcohol syndrome (PFAS), alcohol-related neurodevelopmental disorders (ARND), and alcohol-related birth defects (ARBD) [1,2]. PAE is a substantial cause of mental disability and birth defects in the Western world with an estimated 3–5% prevalence in Europe and North America and over 10% prevalence in South Africa [8]. Several biological and environmental factors are known to influence the effects of alcohol exposure, including the dose of alcohol, the exposure pattern, the duration of exposure, the developmental timing of exposure, the genetic background of the mother and fetus, maternal age, socioeconomic status, nutrition, and interactions with other drugs. Owing to these factors, the phenotype of FASD is complex and the molecular mechanisms of PAE are challenging to study.

The growing amount of evidence suggests that some developmental periods are particularly sensitive to alcohol teratogenesis [9,10]. The preimplantation period lasts for the first two weeks of gestation in humans and 4–6 gestational days (GD) in mice. This period is vulnerable to alcohol exposure [11,12] since the embryo is undergoing rapid developmental changes as the zygote develops into a morula and further into a blastocyst. After the preimplantation period, the blastocyst attaches to the uterine wall and gastrulation begins, which is a time of intense cell differentiation into three germ layers—endoderm, mesoderm, and ectoderm. This period occurs during week three in humans and GD6.5–8.5 in mice. The differentiating cells are particularly sensitive to alcohol exposure, which makes gastrulation one of the most vulnerable developmental periods [13,14,15]. Studies have shown that a single dose of alcohol on any of GD7–9 can produce different craniofacial malformations resembling the features of FASD in mice [16,17,18]. In addition, differences as small as 4 h in the timing of PAE on GD7 resulted in different craniofacial phenotypes [19,20]. Gastrulation is a part of organogenesis, which corresponds to 3–8 weeks of gestation in humans and GD7‒14 in mice. It is a period of continuing cell differentiation and the beginning of rudimentary organ formation, and, therefore, alcohol exposure during organogenesis may produce many features of FASD [21]. These studies have shown that early pregnancy is a sensitive time for alcohol-induced developmental disruptions. Here, we define early pregnancy as the period from fertilization to organogenesis, i.e., weeks 0–8 of gestation in humans and GD0–14 in mice.

### 1.2. Effects of Early Environmental Exposures

Numerous studies have shown that environmental exposures in early pregnancy, and even before pregnancy, can have an impact not only on development but also permanently on a person’s overall health and risk for adult diseases such as cancer, heart disease, obesity, or diabetes [22,23,24,25,26]. David Barker proposed a hypothesis of “Developmental Origins of Adult Health and Disease” (DOHaD), suggesting that the early environment programs fetal development and permanently affects the physiology of the fetus [27]. Although the significant role of the prenatal environment in an adult phenotype seems to be indisputable, the molecular mechanisms underlying these associations or causality of the observed alterations are poorly understood.

The epigenetic variation induced during in utero development has become a strong candidate mediator of the environmental effects. Prenatal exposure to various environmental factors, such as chemicals [28], diet [29], stress [30], obesity [31], tobacco smoking [32], and alcohol consumption [33] has been shown to alter epigenetic modifications in the human and mouse offspring, leading to potential consequences in the phenotype.

The vulnerability of the epigenome in early pregnancy is well demonstrated in studies of prenatal famine and bisphenol A (BPA) exposures. After maternal exposure to famine during the “Hunger winter” in 1944–1945 in the Netherlands, children had a higher risk of having obesity, heart diseases, and diabetes in adulthood [24]. The famine exposure was associated with a decreased DNA methylation of growth promoting the *insulin-like growth factor 2* (*IGF2*) imprinted gene in the whole blood of individuals exposed particularly at early pregnancy [34]. In further studies, DNA methylation changes specific to early pregnancy were also detected in other imprinted genes that are involved in growth as well as in metabolic and cardiovascular disease [35]. The other example, BPA exposure, has been linked to DNA methylation changes and genomic imprinting disruption in prenatally exposed mice [28,36]. Maternal prenatal BPA exposure during the late stages of mouse oocyte development and the early stages of embryonic development (maternal exposure 2 weeks prior to mating until GD9.5 and GD12.5) significantly altered embryonic and placental DNA methylation and the expression of imprinted genes associated with imprinting disorders in humans [37]. In contrast, exposure in later stages of development had no significant effects on the imprinted genes (maternal exposure GD5.5–GD12.5).

**Figure 1:**
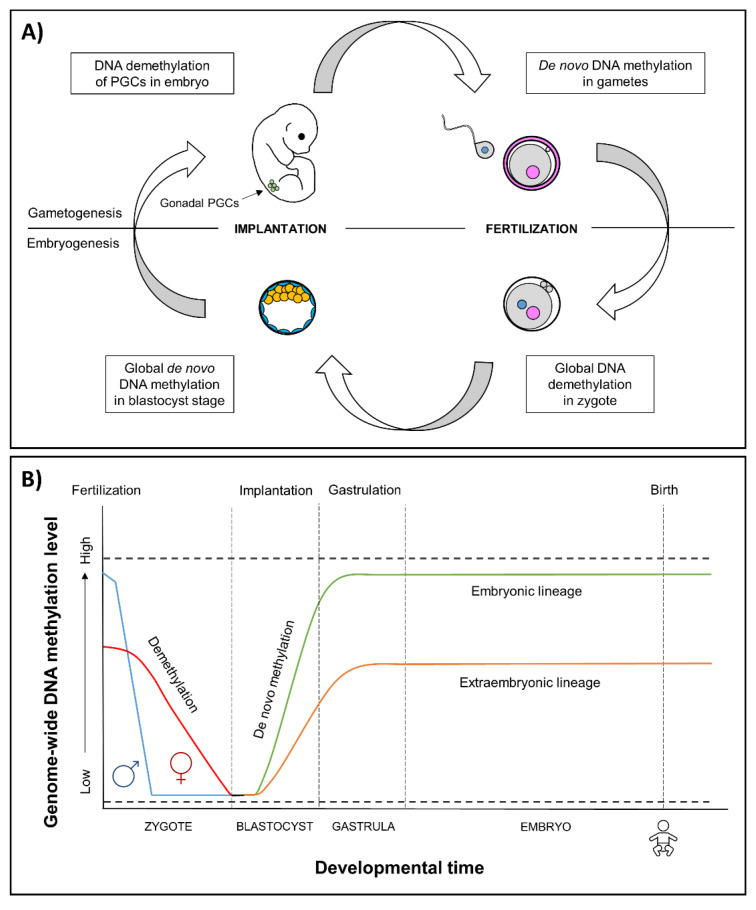
(**A**) Epigenetic reprogramming in the germ line and in somatic cells. Reprogramming occurs in two phases of in utero development, one after fertilization in the preimplantation embryo and the other in the developing gametes of the fetus. In gametogenesis, primordial germ cells (PGCs) become globally demethylated early in development [40]. De novo methylation begins in prospermatogonia in male germ cells during spermatogenesis and after birth in maturing oocytes. In embryogenesis, the epigenetic reprogramming begins after fertilization and continues until the blastocyst stage. Genome-wide de novo methylation occurs during implantation and gastrulation, leading to the formation of different cell types. (**B**) Epigenetic reprogramming in embryos. After fertilization, in the beginning of reprogramming, methyl marks are actively erased in paternal genome (blue line), and passively erased in maternal genome (red line) [41]. By the blastocyst stage, the genome is almost completely hypomethylated [42]. Blastocyst cells divide and differentiate into the following two distinct lineages: the pluripotent inner cell mass, which will become the developing fetus, and trophoectoderm, which forms the extraembryonic tissues. Demethylation is followed by genome-wide de novo methylation during implantation and gastrulation [39], in which the reestablishment of epigenetic marks is more limited in the extraembryonic lineage (orange line) compared to embryonic lineage (green line) [43]. An exception to methylation is made by imprinted genes that remain either methylated or unmethylated throughout epigenetic reprogramming (dashed lines) [39].

The earliest stages of pregnancy and embryonic development comprise a sensitive period of epigenetic reprogramming (Figure 1). During reprogramming, epigenetic marks, especially DNA methylation, are erased and re-established in the early embryo. It provides a regulatory mechanism for the differentiation of cells toward different cell types—reprogramming restores the ability of the zygote to develop into all the different cell types and tissues [38,39]. Environmental-induced disruptions during this period may influence the regulation of developmental genes, resulting in alterations of embryonic development and adverse health outcomes.

## 2. Epigenetic Effects of Early PAE

Several human and animal studies have identified associations between PAE and alterations in epigenetic modifications such as DNA methylation, histone modifications, and non-coding RNAs, including microRNAs (miRNAs) [44,45,46]. However, the molecular mechanisms underlying these alcohol-induced epigenetic effects are still poorly understood. It has been suggested that early PAE may interfere with epigenetic reprogramming and could change the establishment of epigenetic marks. One suggested mechanism is the alcohol-induced reduction in folate, B6, or B12 vitamins in the methionine cycle, which decreases the amount of S-adenosylmethionine (SAM) in the cells [47,48,49]. As SAM is a donor of methyl groups for both DNA and histone methylation, alcohol could change the establishment of epigenetic marks in a developing embryo. Furthermore, acetyl groups from alcohol were rapidly incorporated in histone acetylation in the brain in a mouse model and changes have been observed also in a prenatally alcohol exposed fetal brain [46]. Epigenetic changes in the first embryonic cells could be fixed in persistent cellular memory and mitotically transmitted to different cell and tissue types. Consequently, these alterations may affect gene regulation and depending on the function of the cell types or tissues, they will contribute to the complex phenotype of FASD.

### 2.1. Effects of Early PAE on Epigenome in Mouse Models

Only a few studies have examined the epigenetic effects of in vivo alcohol exposure during the first trimester thus far. The first global methylation study was performed by Garro et al. [50]. Acute alcohol administration twice a day during GD9–11 (in total of five 3 g/kg doses by gavage) resulted in global hypomethylation in DNA methylation profiles in mouse fetuses, potentially by inhibiting *DNA methyltransferase 1* (*Dnmt1*) activity. In later studies, early PAE-induced epigenetic changes were detected in the placenta, but the embryos remained unaffected. Haycock and Ramsay [51] studied the effects of alcohol in GD10.5 mouse embryos and placentas exposed during preimplantation development (2.9 g/kg ethanol injections in females on GD1.5 and GD2.5) and found decreased DNA methylation at the imprinting control region of the *Igf2*/*H19*-imprinted locus in the placentas.

By using a mouse whole-embryo culture, Liu et al. [52] investigated the effects of PAE (88 mM ethanol exposure on GD8.5 throughout 44 h) at early embryonic neurulation. They showed that early PAE causes changes in DNA methylation with associated changes in gene expression and found significant methylation changes in imprinted genes and genes known to have roles in growth, cell cycle, apoptosis, cancer, and olfaction. In this study, specific regions became less methylated and others more methylated in response to alcohol exposure, suggesting that some regions may be more sensitive to the effects of alcohol-induced alterations. In addition, they observed delayed growth and reduced overall growth with significant alteration in the development of the heart, caudal neural tube, brain vesicles, optic system, and limb buds of the embryos treated with alcohol.

By using our mouse model of early PAE (maternal ad libitum ingestion of 10% (*v/v*) ethanol during GD0.5–8.5, approximately 12 g/kg/day), we have shown for the first time that alcohol could affect the adult phenotype by altering the epigenotype of the early mouse embryo [53]. We studied the effects of early PAE by using a metastable epiallele *Agouti viable yellow* (*A^vy^*), which is a dominant mutation of the murine *Agouti* (*A*) locus, caused by the insertion of an intracisternal A-particle (IAP) retrotransposon upstream of the *Agouti* coding exons. The activity of *A^vy^* is variable among genetically identical mice, resulting in mice with a range of coat colors; from yellow to mottled to agouti (termed pseudoagouti) [54]. *A^vy^* is a widely used “biosensor” to study the effects of environmental exposures on the epigenome [55,56]. Our results demonstrated that early PAE increases the DNA methylation level at the *A^vy^* allele and, consequently, alters the coat color of offspring [53]. Furthermore, we found similar changes in gene expression in the hippocampus, olfactory epithelium, and mesodermal bone marrow of adolescent mice, suggesting that changes in gene regulation may have already occurred in the first cells of the embryo [57].

### 2.2. Embryonic Stem Cells as a Model for Early Alcohol-Induced Effects

Most of the early alcohol exposure studies have focused on exposure in in vitro models using undifferentiated or differentiating stem cells. Embryonic stem cells (ESCs) derived from the inner cell mass of a developing blastocyst are pluripotent, having the developmental potential to give rise to all three embryonic germ layers and eventually differentiate into all cells and tissues of an adult organism. The pluripotent identity of ESCs is governed by a network of transcriptional factors, signaling pathways, epigenetic regulators, and structurally open chromatin that holds ESCs in an undifferentiated state. In response to differentiation signals, the transcriptome, epigenome, and chromatin structure in the differentiating cells undergo rapid global changes that silence the pluripotency genes and activate selected lineage-specific genes [58,59].

Since differentiation can be experimentally induced in vitro, and given their indefinite self-renewing capacity, ESCs are a useful model to study cellular identity and early developmental events during embryogenesis. Their environment can be easily manipulated, which makes them a valuable tool to clarify the interaction between genes and environment in the beginning of embryonic development. ESCs have been used in toxicological research and several human and mouse stem cell models have been established to study the early effects of alcohol. As alcohol strongly disrupts the development of the nervous system [60], several models of neural differentiation have also been developed to study the effects of alcohol on early human neural and brain development. To date, studies on ESCs have observed alterations in the proliferation and differentiation properties as well as changes in molecular mechanisms such as in the levels of core pluripotency factors and epigenetic marks [61,62].

### 2.3. Effects of Alcohol on Embryonic Stem Cells and Differentiation

Studies on mouse (mESC) and human (hESC) embryonic stem cells have shown that differentiating cells and developing tissues are more vulnerable to alcohol than already differentiated cells. Arzumnayan et al. [13] showed that 80–84 mM ethanol exposure for 1–6 days affected neither the proliferation nor the expression of pluripotency markers of undifferentiated mESCs, but triggered apoptosis during embryonic body (EB) differentiation. Nash et al. [63], in turn, showed that a low dose of ethanol (20 mM for one week) increases cell proliferation and induces larger colonies, and simultaneously increases cell apoptosis in undifferentiated cells and ethanol-exposed hESC-derived neural progenitor cells. Moreover, Taléns-Visconti et al. [64] showed that ethanol exposure (25 and 50 mM in proliferating or differentiating media) not only impairs neural progenitor cell survival, but also the differentiation of hESCs into neural progenitors and further into mature neurons and astrocytes. Ethanol exposure also induced expression changes of neural differentiation-associated genes and disrupted the actin cytoskeleton of neural progenitors [64].

There is only scarce evidence of the molecular mechanisms behind the alcohol-induced effects on cell differentiation. One potential mechanism is the effects on the signaling pathways that have important roles in the regulation of stem cell differentiation and the control of embryonic development, such as the WNT pathway [65]. Ethanol exposure (25, 50, and 100 mM after definitive endoderm stage until harvesting) has been shown to suppress the early hepatic differentiation of hESC-derived hepatic progenitor cells in a dose-dependent manner by inhibiting WNT as well as the MAPK/ERK pathway [66]. Furthermore, alcohol-induced inhibition in WNT signaling was also observed during human neural stem cell (NSC) differentiation [67] and the cardiac differentiation of mESCs [68,69].

Another possible underlying mechanism of the alcohol-induced effects on differentiation is alterations in the levels of core pluripotency factors *POU class 5 homeobox 1* (*Oct4*), *SRY-box transcription factor 2* (*Sox2*), and *Nanog Homeobox* (*Nanog*) that control the pluripotent state of ESCs [70]. In mESCs, ethanol exposure has been seen to inhibit the loss of core pluripotency markers during both EB (81–84 mM ethanol for 1–6 days) [13] and cardiac differentiation (17.1–51.4 mM for 14 days) [68], suggesting that alcohol may delay the ESC differentiation.

In addition to maintaining pluripotency, the core pluripotency factors are involved in lineage selection during early differentiation events. The dosage of *Oct4* and *Sox2* in differentiating cells determines the lineage commitment—an increased *Oct4/Sox2* ratio induces ESCs to differentiate into mesoendoderm (ME) and, by contrast, a decreased *Oct4/Sox2* ratio into neuroectoderm (NE) [71,72,73]. Interestingly, alcohol has been shown to affect the balance of these lineage specifiers and alter the differentiation trajectory into specific lineages, especially when differentiating into NE. Ogony et al. [74] found that ethanol exposure (25, 50, and 100 mM for 0–6 days) increases the expression of *Oct4* in a dose- and time-dependent manner, elevates the overall *Oct4/Sox2* ratio, and misleads the cells into an ME cell fate during ESC differentiation into NE. Furthermore, they investigated the effects of ethanol (100 mM for 0–6 days) on the *Oct4/Sox2* ratio as well as on the expression of other downstream genes involved in pluripotency, differentiation, and signaling during the mESC differentiation into NE [62]. Alcohol exposure was shown to downregulate 19 pluripotency genes and upregulate 14 differentiation-associated genes. The changes in the NE differentiation-associated genes altered the overall gene expression dynamics, which could explain the different trajectory observed during the differentiation of alcohol-exposed cells [62].

**Figure 2:**
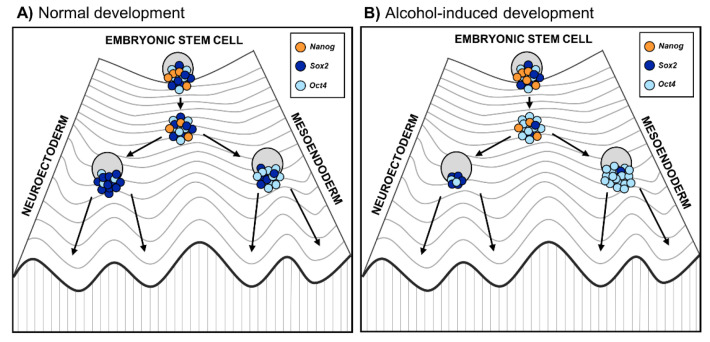
Embryonic stem cell differentiation into germ layers during (**A**) normal embryonic development and (**B**) altered development due to prenatal alcohol exposure. The Waddington’s epigenetic landscape reflects the differentiation of cells, where the fate of stem cells depends on their history as well as developmental and environmental inputs [76]. On the highest hill are ESC progenies that progress toward differentiation. Changes in epigenetic modifications in ESC progenies lead to a reconfiguration of the core transcription factors Oct4, Sox2, and Nanog, represented as a Waddington’s landscape [77]. Coming down to the hill, stem cells first reach a plateau with low Nanog expression that enables them to respond to differentiation signals. (**A**) During normal stem cell differentiation, higher Oct4 expression induces stem cells toward the mesoendoderm progenitor cell fate, while higher Sox2 drives stem cells toward the neuroectoderm progenitor cell fate; (**B**) During alcohol-altered stem cell differentiation, alcohol affects the balance of the core pluripotency factors, especially Oct4/Sox2 ratio [74]. Therefore, alcohol may reprogram lineage specification favoring excess of Oct4 relative to Sox2 and forcing stem cells down the differentiation trajectory away from neuroectoderm.

These results collectively suggest that alcohol reprograms the lineage specification by changing the balance of core pluripotency factors *Oct4* and *Sox2,* thereby forcing ESCs away from neuroectodermal cell fate. This is in line with previous animal studies in which alcohol exposure during gastrulation caused neural progenitor pool reduction [15] as well as long-term effects on the forebrain and mature brain stem nuclei structures [75]. The effect of alcohol on lineage specification is well illustrated by Waddington’s epigenetic landscape model, which describes how development is unidirectional, meaning that embryonic stem cells develop into a mature differentiated state [76]. If alcohol disrupts the balance of the core pluripotency factors, it could force stem cells down the differentiation trajectory away from neuroectoderm (Figure 2). This misguidance could result in developmental delay and defects of the nervous system underlying FASD phenotypes.

### 2.4. Epigenetic Effects of Alcohol on Embryonic Stem Cells and Differentiation

The effects of alcohol on the epigenome have been mainly studied in differentiated cells such as NSCs and heart progenitors rather than in undifferentiated ESCs or specifically in gastrulation. However, the results have shown associations between ethanol exposure and alterations in epigenetic mechanisms.

Alcohol-induced changes in the DNA methylation of ESCs and differentiating cells were studied in a genome-wide DNA methylation sequencing analysis, which revealed significant alterations in the methylation and transcriptomic profiles of ethanol-treated (20 mM for 24 h) undifferentiated hESCs, leading to reduced pluripotency, and also ethanol-treated differentiated EBs [61]. A higher global hypermethylation in undifferentiated ESCs than in EBs was observed at the promoter regions, suggesting that the methylomes of undifferentiated ESCs are more prone to alcohol-induced effects than the methylomes of already differentiated cells. The changes in transcriptomic of undifferentiated hESCs and EBs were associated with oxidative stress, metabolic processes, and neuronal properties [61].

DNA methylation changes have also been detected in ethanol-exposed mouse NSCs. Hicks et al. [78] focused on promoter regions and found that ethanol exposure (86.8 mM for 48 h) prolonged the total length of the cell cycle, increased the activity of *Dnmt1* and induced hypermethylation of several cell cycle genes. Considerably, the results of the increased activity of *Dnmt1* in NSCs contradict the findings of Garro et al. [50], who showed that PAE decreases *Dnmt1* activity in fetal mice. Moreover, Zhou et al. [79] found that binge-like ethanol exposure (88 mM for 6 h) delayed the migration, neuronal formation, and growth of rat NSCs and prevented the methylation of genes associated with neural development, eye development, and developmental disorders during the reprogramming of quiescent NSCs into differentiation. Ethanol exposure (70 mM for 48 h or 8 days) during NCS differentiation has also been shown to increase the expression of *methyl CpG binding protein 2* (*Mecp2*), an important epigenetic factor in the brain, in association with decreased DNA methylation and increased hydroxymethylation at its regulatory elements [80].

The knowledge of alcohol´s effects on the chromatin structure in ESCs is scant, but a few studies show alterations of histone modifications in NSCs [81,82] and heart progenitor cells [83,84]. Veazey et al. [81,82] studied the effects of alcohol on histone modifications (H3K4me3, H3K9me2, H3K9ac, H3K27me3) at specific genes associated with development in fetal mouse NSCs and showed that ethanol exposure (35 and 52 mM for 3 days and 70 mM for 5 days) exhibits significant alterations in the chromatin structure. The effects were dependent on the gene of interest and the dose of ethanol as well as whether the cells had recovered from the treatment before harvesting [82]. Interestingly, after the recovery, most of the changes were observed in histone marks associated with a repressive chromatin structure (H3K9me2 and H3K27me3). These changes were also consistent with the repressed chromatin structure observed in the in vivo mouse model (ethanol exposure on GD7), correlating with the development of craniofacial and central nervous system defects as well as a decreased expression of genes that are associated with development and epigenetic reprogramming [82]. The ethanol exposure (50 and 200 mM for 24 h) of mouse heart progenitor cells has been shown to increase H3K9 acetylation and change the expression of the genes involved in heart development [83,84].

Extensive alcohol-mediated changes in miRNA and miRNA-target gene expression have been observed in utero that can be detrimental to the developing embryo [85]. These alterations are mainly studied in mouse neural progenitor cells in the early embryonic stage. For instance, Sathyan et al. [86] found that ethanol (70 mM for 5 days) suppresses the expression of miRNAs (miR-21, miR-335, miR-9, and miR153) in cerebral cortical neuroepithelial precursors. Later, they reported that exposure also suppressed miR-140-3p in cultured neurospheres, suggesting that relatively small teratogen-induced changes in miRNAs during stem cell differentiation can result in long-lasting deficits in brain function [87].

## 3. Effects of Early PAE-Induced Epigenetic Alterations on Phenotype

Although PAE has been associated with alterations in the epigenome, the causal molecular mechanisms between the alcohol-induced epigenetic alterations and the adult phenotype are still unknown. Any epigenetic changes during early or even whole pregnancy that alter gene regulation and result in a human FASD phenotype are not yet revealed. Due to the limited number of human studies, the only evidence of a connection between early alcohol-induced epigenetic alterations and the phenotype is thus far based on studies using animal models.

The hypothesis of the early epigenetic origin of alcohol-induced disorders is supported by studies that have found epigenetic changes associated with the phenotype or comorbidities of FASD in various tissues. In GD9 mice embryos, acute PAE (5.8 g/kg, intragastric intubation) caused a subtle decrease in the DNA methylation of *Igf2*’s differentially methylated region in embryonic tissue, which led to a decrease in *Igf2* gene expression [88]. These gene expression changes were associated with skeletal malformations that resemble those found in individuals with FAS. Moreover, studies on GD7.5–16.5 mouse embryos found that PAE (56% (*v/v*) ethanol by gavage) is linked, in addition, to global H3K9 hyperacetylation, also *GATA binding protein 4* (*Gata4*) promoter histone H3K9 hyperacetylation, which leads to *Gata4* overexpression in cardiac tissue [89,90]. *Gata4* is an essential transcription factor during heart development, and it is presumed that alterations of *Gata4* expression can affect the epigenetic regulation of embryonic development and contribute to congenital heart disease in children [90]. Subsequently, studies have shown that PAE (56% (*v/v*) ethanol by gavage or intragastric administration) increases the mRNA expression of developmental genes and also causes the hyperacetylation of H3K14 in the fetal hearts of mice [91,92].

Although we have shown for the first time that early PAE could affect the epigenome of an early embryo and, consequently, an adult phenotype by using the metastable epiallele *A^vy^* [53], it is not a normal allele and, as such, cannot prove a link between early epigenetic alterations, gene expression, and the phenotype. However, adult PAE offspring without *A^vy^* allele were examined and the phenotype was reminiscent of human FASD with craniofacial dysmorphology, postnatal growth restriction [93], and structural changes in the central nervous system [57,94]. Interestingly, this same mouse model, with relatively moderate PAE, is associated with decreased DNA methylation in the *solute carrier family 17* (*Slc17a6*) promoter region, which plays a role in neurotransmission, synaptic plasticity, and cognition and encodes vesicular glutamate transporter (VGLUT2) in mice [95]. *Slc17a6* showed increased mRNA levels together with decreased promoter DNA methylation, decreased VGLUT2 protein levels, and increased H3K4me3 in the alcohol-exposed (GD0.5–8.5) adult male hippocampus. Furthermore, 15 ethanol-sensitive miRNAs were found in the hippocampus, three of which (miR-135a, miR-135b, and miR-467b-5p) were also differentially expressed in serum, suggesting that serum expression could be used as a biomarker for expression levels in the hippocampus. The study indicates that PAE can cause long-term deregulation of brain DNA methylation, histone modification, and gene expression. A comprehensive battery of behavioral tests was conducted for the adult mice, and persistent and long-lasting alterations in behavior, including human FASD-phenotype-associated hyperactivity, were observed [96].

## 4. Conclusions

Several animal and stem cell studies have shown that early alcohol exposure is capable of altering epigenetic marks as well as affecting cell differentiation, embryonic development, and the adult phenotype. Based on these studies, alcohol-induced epigenetic perturbations are subtle changes, resulting in shifts toward increased or decreased gene expression. However, these subtle changes can disturb critical developmental processes in which optimal gene function and adequate timing is required. Indeed, alcohol exposure in gastrulation can affect the developmental trajectory and turn cell fate away from ectoderm in differentiating mESCs, although the epigenetic mechanisms are still unknown. It would be attempting to hypothesize that those alcohol-induced epigenetic changes in the first cells are transmitted mitotically to different cell and tissue types, consequently contributing to the developmental abnormalities in the complex FASD phenotype, but any causal link is not yet known.

The studies related to early PAE have mainly been performed by using mESC cultures or rodent models with variable exposure periods, doses, and methods. Due to this variability, the results are often scattered and discordant—even conflicting—and, thus, difficult to construe. Therefore, more systematic research by using biological material as well as developmental information derived from both humans and animals are needed. In the future, a growing number of genome-wide studies, novel research models such as placental organoids, and transition from bulk DNA to single cell analysis, will dramatically increase our understanding of the epigenome’s role in the molecular alterations caused by PAE. Both the sensitivity of the epigenome in early pregnancy and the mitotically heritable nature of the epigenetic marks make the early developmental period particularly intriguing in the pursue of clarifying the etiology of complex developmental disorders. This will help us understand the interaction between genome and environment in early development and the effects of these interactions on our phenotypes in health and disorders.

## Data Availability

No new data were created or analyzed in this study. Data sharing is not applicable to this article.
